# Highly Effective Sensitizers Based on Merocyanine Dyes for Visible Light Initiated Radical Polymerization

**DOI:** 10.3390/polym12061242

**Published:** 2020-05-29

**Authors:** Beata Jędrzejewska, Grażyna Wejnerowska

**Affiliations:** Faculty of Chemical Technology and Engineering, UTP University of Science and Technology, Seminaryjna 3, 85-326 Bydgoszcz, Poland; Grazyna.Wejnerowska@utp.edu.pl

**Keywords:** merocyanine dyes, photoinitiators, radical polymerization

## Abstract

The 5-(4-substituted-arylidene)-1,3-dimethylpyrimidine-2,4,6-triones were tested as visible light sensitizers for phenyltrialkylborate salts applied to initiate polymerization processes. The initiation occurs as a result of photoinduced electron transfer from the borate salt to the merocyanine dye. The main factor that facilitates the step of the reaction is the free energy change for electron transfer. Its value is favorable according to the reduction properties of the dyes influenced by the type of amino groups and the oxidation potentials of the borate salts. The observed bleaching of the dyes during photopolymerization affects the yield of both the alkyl radical and sensitizer-based radical formation and thus the efficiency of the photopolymerization.

## 1. Introduction

Photoinitiated polymerization uses the energy of light for rapid conversion of monomers to solid polymeric products. The term photopolymerization implies that light absorption is crucial for the initiation step of a radical, cationic, or anionic chain reaction producing a macromolecule. The absorption of one photon may start the reaction of up to 10^5^ monomeric units [1,2]. The radicals responsible for an initiation of radical polymerization may be generated by direct photolysis of a photoinitiator (PI) as a result of bond dissociation or in bimolecular processes [1,3,4]. In the first case, UV light is usually needed to initiate the unimolecular fragmentation of a precursor. To extend this concept far into the visible region of the spectrum, a sensitizer needs to be added to the initiator. Thus, the panchromatic sensitization of polymerization requires the presence of a dye as a primary absorber which forms the photoinitiating system with a suitable co-initiator. In that case free radicals are formed as a result of bimolecular processes. For example, sensitization that is useful in the blue region, may involve a bimolecular electron-transfer reaction between an excited dye and an electron donor to form reactive intermediates, a radical anion, and a radical cation. Subsequent proton transfer results in radicals that are capable of initiating polymerization [1,3,4].

Oster [5] first described the process of free radical generation by dyes photoreduced in the presence of suitable reductants like ascorbic acid and triethanolamine. The systems included acridine and xanthene dyes. Since then, many other dyestuffs have been designed as photosensitizers [6] that extend the spectral range of the photopolymerization to the visible region [7,8,9,10,11,12,13,14] because this light is cheap, safe and possesses higher penetration in comparison to UV light [15].

**Scheme 1:**
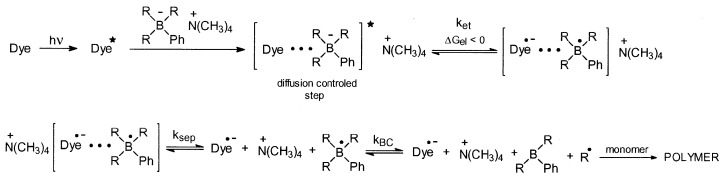
The processes that may occur during the radical photoinduced polymerization with the use of dye-borate initiators; *k*_BC_ denotes the rate constant of carbon–boron bond cleavage, *k*_sep_ describes the rate constant of the separation step, and *k*_et_ is the rate constant of the electron transfer from borate anion to dye.

Such systems are of interest because of their use in many special applications, such as dental filling materials, photoresists, printing plates, highly pigmented coatings, holographic recordings, and nanoscale micromechanics [5]. Depending on the nature of the dye involved, two distinct types of sensitization need to be considered: the electron transfer from the co-initiator to the excited, photoreducible dye and the electron transfer from the excited, photooxidizable dye to the co-initiator [4]. The initiating radicals are subsequently formed from cleavage of the oxidized or reduced form of the co-initiator. In photoreducible sensitization, the commonly used co-initiators are: N-phenylglycine [8], phenylthioacetic acid [16], amine [17,18], and alkyltriphenylborate salts [19,20], whereas *N*-alkoxypyridinium salts [21,22] and the 2,4,6-tris(trichloromethyl)-1,3,5-triazine derivatives are applied as co-initiators in photooxidizable sensitization [9,23]. For example, in photoreducible sensitization one electron oxidation of alkyltriphenylborate salts yields an alkyltriphenylboranyl radical that undergoes carbon–boron bond cleavage giving an alkyl radical and neutral molecule of triphenylborate [3]. The general mechanism of the dye-sensitized photodecomposition of phenyltrialkylborate salts [3] is presented in Scheme 1.

The search for new candidates in photoinitiators’ design is ongoing and a major challenge is to develop compounds active under milder conditions than the ones which require employing traditional UV irradiation sources [10,24]. This is due to a growing emphasis on resource and energy conservation, as well as environmental concerns. Commonly used in industry, photoinitiators operate under high light intensities from high-energy UV sources which give rise to safety and financial concerns. The development of photoinitiators that absorb strongly in visible light region allows the academic and industrial communities to eliminate these drawbacks, for example, by using inexpensive irradiation setup like low-power light-emitting diodes (LEDs) and diode-pumped solid state (DPSS) lasers [25]. In recent years, relatively many families of visible PIs usable in photoinduced polymerization reactions have been developed [25]. They were designed not only for application in conventional areas (coatings, inks, and adhesives) but also high-tech domains such as optoelectronics, laser imaging, stereolithography, three-dimensional (3D) printing, and nanotechnology [26]. It has become evident that compounds designed for the above-mentioned applications should exhibit excellent light absorption properties and be able to be incorporated in photoinitiating systems. The strategy for photoinitiator synthesis follows either the preparation of new chemical skeletons or modification of existing structures, as well as the use of new additives [25]. Regardless, each PI should exhibit excellent absorption of visible light, efficiently interact with additives (e.g., electron donors or electron acceptors), and reveal long-lived singlet (or triplet) state lifetimes. In this context, merocyanine dyes based on barbituric acid were identified as ideal candidates as photoinitiators of free radical polymerization reaction under air, under low intensity of light sources, and upon visible light exposures, because they fulfill the aforementioned requirements. 

In general, merocyanines belong to the class of neutral unsymmetrical polymethine dyes. Their structure is based on push-pull architecture comprised of two fragments—the nitrogen donor and oxygen acceptor—on opposite ends of the ethylene or polyethylene bridge. The dyes have found wide application in various areas of science and technology including nonlinear optics, solar and hydrogen energy, nanotechnology, and laser technology [27,28,29,30]. They are used as optical sensors, spectral sensitizers for silver halide photography, recording medium in optical disks, photosensitizers for photodynamic therapy, and radiation sensitizers for solid tumor treatment [31,32,33,34]. 

Despite a variety of photoinitiators active in the UV–Vis region, there are only a few reports that deal with photopolymerization initiated by systems containing barbituric acid derivatives. Kitamura et al. [35] studied the sensitizing abilities of near infrared-absorbing (NIR) non-ionic cyanine dyes in which barbituric acid was conjugated by a double bond at the center of a polymethine chain. Their sensitizing ability was tested in the presence of 2,4,6-tris(trichloromethyl)-*s*-triazine (TCT) as a radical generator. The authors revealed that the barbiturate-functionalized non-ionic cyanine dyes showed a high sensitizing ability in radical polymerization, which is due to a high efficiency of the photoinduced electron transfer reaction and generation of many radicals. 

Similar photoinitiator systems being a combination of a NIR-sensitizer and a radical initiator were investigated in different multifunctional monomers by Strehmel research group [29].

Another group of barbiturate-functionalized dyes tested as visible light photoinitiators was developed by Fouassier and Lalevée research group [36]. The push-pull dyes were based on (thio)barbituric acid derivatives and a carbazole moiety connected by a vinyl group. The main advantages of these sensitizers is the possibility of working in both an oxidative cycle (dye/diphenyl iodonium salt/*N*-vinylcarbazole system) and a reductive cycle (dye/amine/alkyl halide system). Furthermore, the dyes partly behave as organic photocatalysts. The authors discussed the contrasting behavior of the thiobarbituric vs. barbituric acid derivatives. They found that the ring-opening polymerization (ROP) of epoxides and radical polymerization (RP) of acrylates were easily promoted.

Barbituric acid was also used in dye-linked photoinitiators in which a radical generating part was covalently bonded to a photosensitizing dye chromophore. Such systems have shown to be very useful as free-radical sources. For example Kawamura et al. [37] prepared a dye-linked initiator consisting of thiobarbituric acid and a substituted bis(trichloromethyl)-1,3,5-triazine initiator. They revealed that photoinitiating efficiency of dye-linked initiators in acrylates photopolymerization is higher than in a system containing the dye and triazine mixture.

The (thio)barbituric acid derivatives were also used to reveal their radical-scavenging activities. The measurements were based on the induction period (IP) method for polymerization of methyl methacrylate (MMA) initiated by thermal decomposition of 2,2′-azobisisobutyronitrile (AIBN). Kadoma and Fujisawa tested eight 2-thiobarbituric acid derivatives containing different substituents of nitrogen and carbon atoms. They found, based on IP data, that the type of substituent and its location change their properties. Some of them may act as a weak antioxidant, an inhibitor, while others as a prooxidant. The 5,5-dimethyl-thiobarbituric was found to be the most potent inhibitor. The influence of the thiobarbituric acid derivatives on the average molecular weight of the polymers formed from MMA was discussed as well [38].

Moreover, Chern et al. investigated both the isothermal and non-isothermal polymerizations of *N*,*N*’-bismaleimide-4,4-diphenylmethane with barbituric acid [39]. The authors showed that the polymerization was governed by the competitive Michael addition reaction and free radical polymerization mechanism.

Our main task, in the present work, is to determine the possibility of application of thirteen representatives of 5-(4-substituted-arylidene)-1,3-dimethylpyrimidine-2,4,6-triones as sensitizers in visible light photoinitiating systems. The tested systems, composed of the dyes and borate salts used as electron donors, generate free radicals by photoreducible dye sensitization mechanism. Through this photochemical approach, radicals able to initiate the free radical polymerization are generated by an electron transfer reaction between the excited state of the merocyanine dye and the phenyltriethylborate salt.

The designed sensitizers are based on classical push-pull architecture for which the transfer of charge from nitrogen donor to oxygen acceptor through the polyene chain gives rise to the deep coloration. This effect depends on both chain length and the nature of the donor and acceptor groups. The merocyanines under study differ by the electron releasing properties of a substituent (alkyl or aryl amino group) present in the aromatic moiety (for structure see Table 1).

**Figure 1:**
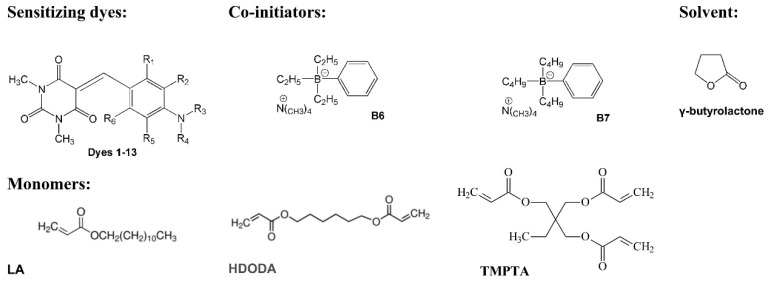
The general structure of dyes tested as light absorber and borate salt used as co-initiator in photoinitiated polymerization of acrylate monomers.

The goals of this work are to check how the shape of the alkylamino group and the relative orientation of the nonbonding electron pair, with respect to the phenylene, affect the photophysical properties of the merocyanines, which have an impact on their photoinitiating abilities: (a) absorption and fluorescence properties including spectral range, molar absorption coefficient, and singlet state energy; (b) photostability; (c) reduction potential and as a consequence the free energy of activation of the electron transfer process. Thus, we evaluate the influence of the amino moiety on the photoinitiating abilities of the tested systems for acrylate polymerization. The chemical structures of the photoredox system as well as the solvent and monomers used are shown in Figure 1.

## 2. Materials and Methods 

**Scheme 2:**
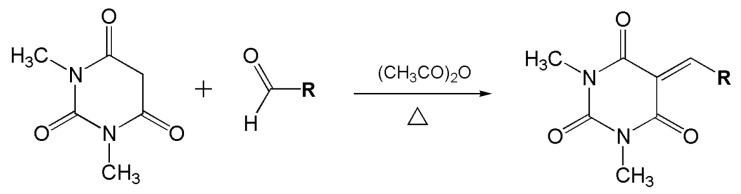
General route for the synthesis of 5-(4-substituted-arylidene)-1,3-dimethylpyrimidine-2,4,6-triones. For **R** see substituents in Table 1.

The merocyanine dyes, 5-(4-substituted-arylidene)-1,3-dimethylpyrimidine-2,4,6-triones, were synthesized in our laboratory according to the method described by Würthner and Yao [32]. The general route for their synthesis is presented in Scheme 2. The substituent structures in 5-(4-substituted-arylidene)-1,3-dimethylpyrimidine-2,4,6-triones together with abbreviations used are shown in Table 1. All solvents (reagent or spectroscopic grade, ethyl acetate (EtOAc), *γ*-butyrolactone) and monomers (lauryl acrylate (LA), 1,6-hexanediol diacrylate (HDODA), and trimethylolpropane triacrylate (TMPTA)) were purchased from Aldrich Chemical Co. and used without further purification. 1,3-Dimethylbarbituric acid was also purchased from Aldrich Chemical Co., acetic anhydride ((CH_3_CO)_2_O) was purchased from Avantor Performance Materials Poland S.A., and aldehydes for dye synthesis were obtained in our laboratory.

Chromatography. Gas chromatography (GC) experiments were done using a HP 5890 (Hewlett-Packard, CA, USA) gas chromatograph, equipped with a split/splitless injector and flame ionization detector (FID) system. Analytes were separated with a ZB-5 (5% phenyl-95% dimethylpolysiloxane) column (Zebron, Phenomenex, CA, USA), 30 m × 0.53 mm I.D., with a film thickness of 1.50 μm. Helium was used as a carrier gas at a constant flow rate of 1.2 mL/min. The amount injected was 1 μL. The temperature of the injector and the detector was 250 °C. The oven temperature was 50 °C for 4 min, and then programmed to 250 °C at 10 °C/min and held there for 0 min. The reaction mixture consisted of tetramethylammonium phenyltriethylborate:5-(4-*N*,*N*-dimethylamino-arylidene)-1,3-dimethylpyrimidine-2,4,6-triones (**1**):radical trapping agent in a 1:1:10 ratio. As radical trapping agents, we used methyl acrylate. 

Spectral Measurements. Electronic absorption spectra for ca. 10^−5^ M dye solution in ethyl acetate (EtOAc) were recorded at room temperature on a Shimadzu UV–Vis Multispec-1501 spectrophotometer (Kyoto, Japan). Emission spectra were obtained from a Hitachi F-7100 fluorescence spectrophotometer (Tokyo, Japan). The solution concentration was ca. 10^−6^ M.

Electrochemical Measurements. Cyclic voltammetry method was used to determine reduction potentials of the dyes and oxidation potentials of the borate salts. The electrochemical measurements were performed on an Electroanalytical Cypress System Model CS-1090 (Lawrence, KS, USA) equipped with three-electrode setup. A platinum 1-mm disc electrode was applied as a working electrode whereas a platinum wire and Ag/AgCl were used as auxiliary and reference electrodes, respectively. A 0.1 M solution of tetrabutylammonium perchlorate in anhydrous acetonitrile, purged with argon prior to measurement, was employed as a supporting electrolyte.

Photobleaching. The photobleaching experiments were carried out in quartz cells (1 cm in width). A 4.3 mL solution of the dyes in ethyl acetate (or ethyl acetate:*N*-methyl-2-pyrrolidinone mixture 5:1 *v*/*v*) was stirred and irradiated with a DPSS blue laser from Shanghai Dream Lasers at 457 nm (light power of 50 mW). The irreversible bleaching of the dyes at the absorption peak was monitored as a function of time. Thus, the absorption spectra were recorded at different times during the irradiation. The concentration of the dye in solution was ca. 1.5 × 10^−5^ M. 

Polymerization measurements. The sensitizing properties of the merocyanine dyes were evaluated by measuring photopolymerization exotherms using a photo-differential scanning calorimetry (DSC) apparatus constructed based on a TA Instruments DSC 2010 Differential Scanning Calorimeter (New Castle, DE, USA). The two-component photoinitiating systems contain dyes as sensitizers at a concentration corresponding to an optical density of 0.25 or 2, respectively, at the excitation wavelength (488 nm) and an electron donor, tetramethylammonium phenyltriethylborate (B6), at a concentration of 0.05 M. In addition, the polymerization solution was composed of 1 mL of *γ*-butyrolactone (solvent) and 9 mL of 2-ethyl-2-(hydroxymethyl)-1,3-propanediol triacrylate (TMPTA) which was chosen as a monomer for the free radical polymerization. The reference formulation did not contain the electron donor (borate salt). The reaction was conducted in an open aluminum pan with a diameter of 5 mm and a thickness of 2 mm which contained approximately 0.035 ± 0.002 g of formulation components. The polymerizing mixture was not deaerated before curing. The measurements were carried out at ambient temperature and performed at least three times. A 488 nm line of an argon ion laser (Melles Griot 43 series model) with intensity of light of 100 mW/cm^2^ was used as the light source. The average power of visible light irradiation was measured by a Coherent Model Fieldmaster Power Meter (Göttingen, Germany).

The reaction heat liberated in the polymerization was directly proportional to the number of vinyl groups reacting in the system [40]. The conversion percent of the acrylate double bond (C%) was calculated by integrating the area under the exothermic peak and comparing it to the theoretical reaction heat for 100% conversion. For an acrylic double bond, Δ*H_p_^theor^* = 78.2 kJ/mol [41]. The rate of polymerization (*R_p_*) was directly related to the heat flow dH/dt, whereas the quantum yield of polymerization *Φ_p_* was defined as the number of polymerized double bonds per absorbed photon. Detailed information about calculation of polymerization rate (*R_p,max_*), conversion of the acrylate double bonds (*C*), and polymerization quantum yield (*Φ_p_*) may be found in our previous papers [19,20] as well as in the work of Avci, Nobles, and Mathias, [42]. 

## 3. Results

**Figure 2:**
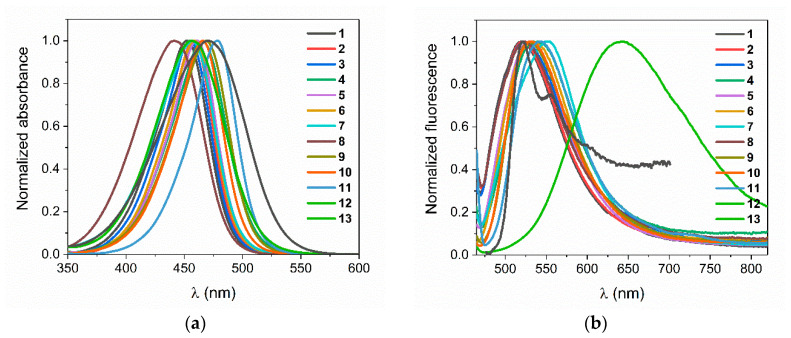
Normalized electronic absorption (**a**) and one-photon fluorescence (**b**) spectra of **1–13** in EtOAc at room temperature (RT); *λ*_Ex_ = 450 nm.

The electronic absorption spectra of the tested compounds had one main absorption band of high intensity with a maximum in the wavelength range of 442–477 nm in EtOAc (Figure 2a). This means that the dyes were suitable to absorb light emitted by laser at 457, 473, and 488 nm. Moreover, they characterize broad structureless emission spectra with maximum at about 521–642 nm (Figure 2b). The relatively large Stokes’ shift (ca. 2480–6354 cm^−1^ in EtOAc) exhibited is common to π-conjugated donor-acceptor compounds and attributed to the charge-transfer character.

Basic photophysical data determined based on electronic absorption and fluorescence spectra measured in ethyl acetate (EtOAc) are collected in Table 1.

### 3.1. Photostability

**Figure 3:**
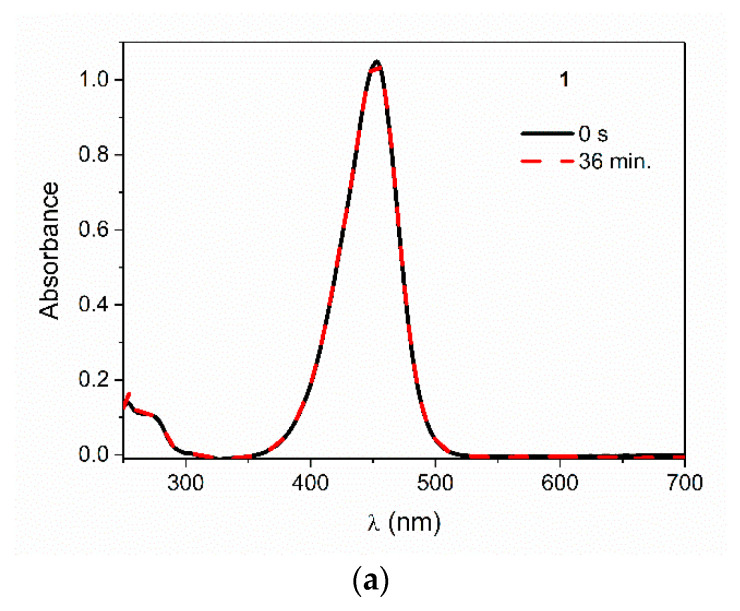

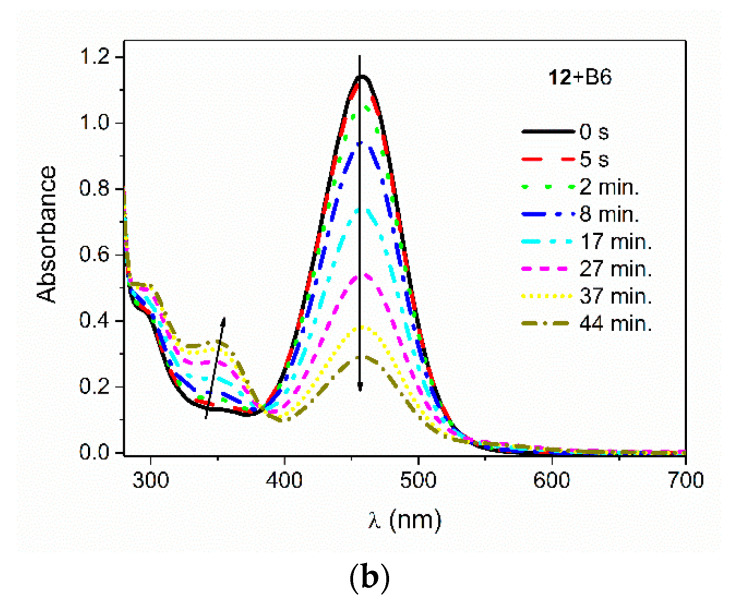
Electronic absorption spectra obtained upon irradiation of a system comprising an air-saturated solution of (**a**) dye **1** (1.5 × 10^−5^ M) and (**b**) dye **12** (3 × 10^−5^ M) in the presence of **B6** (3 × 10^−3^ M) dissolved in the mixture ethyl acetate: 1-methyl-2-pyrrolidone (20:4, *v*/*v*). Spectra were collected before and after laser irradiation (line 457 nm, *I*_0_ = 50 mW).

The photostability of the dyes in ethyl acetate solution was checked prior to photopolymerization experiments. Negligible photobleaching (see Figure 3a) was observed under direct irradiation with 457 nm light during periods of time longer than those required for photopolymerization (so that the sample is cured) (see Figure S3 in Supplementary Materials). High stability of the dye in the solution during irradiation suggests that these compounds are promising photosensitizers for visible curing.

However, photobleaching of the dye was found upon irradiation of the solution containing an electron donor (borate salt) (see Figure S2 in Supplementary Materials). The absorption spectra of the combination of **12** dye and **B6** borate salt before and during irradiation are shown in Figure 3b. Decay of the dye absorption at 456 nm is accompanied by the growth of the bands at ~350 nm. The same effect was observed during photobleaching of the dye/**B7** system. The isosbestic points (Figure 3b) indicate direct conversion of the dye to the product, which is stable. The photobleaching quantum yields of the **12** dye (*Φ_bl_*) in the presence of **B6** and **B7** borate salt are equal 0.0339 and 0.0351, respectively. These data clearly indicate the group of dyes shows high color lost. The reaction took place in the solution containing 3 × 10^−3^ M **B6** and 3 × 10^−5^ M dye, which may indicate formation of the radical anion of the dye (i.e., **12**).

**Figure 4:**
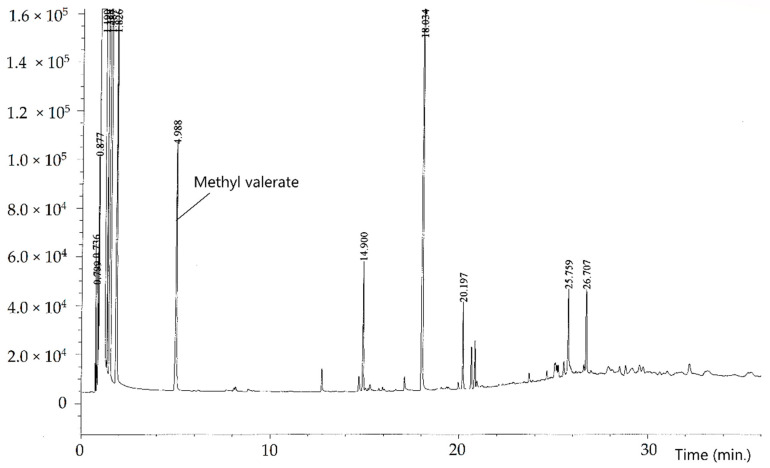
Gas chromatogram of the reaction mixture of tetramethylammonium phenyltriethylborate, methyl acrylate, merocyanine dye, and acetonitrile after laser irradiation (line 457 nm, *I*_0_ = 50 mW).

To explain this effect, the products of photoinduced one-electron oxidation of phenyltriethylborate anion by the excited state of merocyanine dye were characterized chromatographically. The goal of the experiment was to understand whether one of the products of the photoreaction is an ethyl radical. The reaction mixture consisted of NMe_4_B(Ph)Et_3_ as the source of ethyl radical, methyl acrylate as a radical trapping agent, dye **1** as a light absorber, and acetonitrile as a solvent. The gas chromatogram of the reaction mixture is represented in Figure 4.

As we expected among the main signals, there was a peak at 4.988 min belonging to the product of the addition of ethyl radical to the methyl methacrylate. The retention time was confirmed by an injection of the standard solution of methyl valerate (Et–CH_2_–CH_2_–COOMe). The gas chromatography–mass spectrometry (GC-MS) studies also showed mass spectral signal with molecular mass assumed to methyl valerate. In the control experiment without either dye or borate salt, no signal with a retention time of 4.9 min was found. The results clearly indicate that methyl valerate is produced if both borate and dye are present in the reaction mixture. This confirms the formation of ethyl radical from tetramethylammonium phenyltriethylborate by photoinduced one-electron oxidation. 

### 3.2. Photoinitiating Polymerization Properties

A number of factors [1,7,19,23,43,44,45] affects the kinetics of polymerization of multifunctional monomers. A very important one is the steady-state assumption that the concentration of the radical intermediates stays constant throughout the polymerization. In other words, the initiation and termination rates are equal. However, it is only valid at the conversion of a monomer not exceeding 1–3%. Other factors are connected with the rates of initiation, propagation, and termination which are time diffusion controlled and change during polymerization [46,47,48]. 

**Figure 5:**
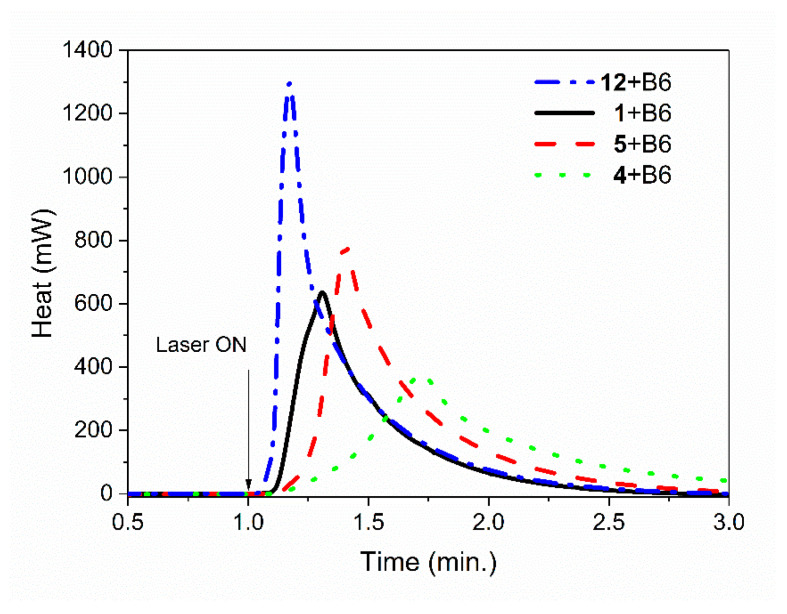
Family of kinetic curves recorded during the measurements of the heat flow emitted during the photoinitiated polymerization of trimethylolpropane triacrylate (TMPTA) initiated by merocyanine borate systems marked in the figure. The borate salt concentration was 5 × 10^−2^ M, optical density of the dyes was 0.25 at an excitation wavelength, *I*_0_ = 100 mW/cm^2^.

Typical polymerization kinetic curves obtained by photo-DSC for the studied merocyanine dyes–borate salt systems are shown in Figure 5 whereas the data *R_p max_* are collected in Table 2. 

The photo-DSC profiles of the TMPTA polymerization recorded for the dye–borate couples are typical for multifunctional monomers and indicate high efficiency of the photoinitiator systems in the initiation of the chain reaction (see Figure S3 in Supplementary Materials). Directly upon irradiation the rate of polymerization increases because of gelation which limits the diffusion and mobility of both macroradicals and pendant double bonds. As a result, the radical termination rate slows down, and an accumulation of radical species occurs leading to autoacceleration. However, further formation of a three-dimensional structure restricts the monomer mobility and the diffusion starts to control the propagation and termination steps as well. Thus, the overall polymerization rate begins to decrease and eventually the chain reaction stops due to trapping of the radicals in the polymer network [47,48].

The efficiency of the polymerization initiated by merocyanine dye–borate salt systems was assessed from the heat flow during the irradiation (Figure 5). In the case of these photoredox pairs, the conversion of the monomer into polymer was in the range of 30% to 70%, while the quantum yields of photopolymerization oscillated between 4.2 and 207.4. According to the polymerization rates data, the photoinitiation efficiency of the tested systems depends on a character of the dialkylamino substituent in the electron donating part of the molecule. The best photoinitiating abilities exhibit the photoredox pairs possessing diphenylamino (**12**) substituent in the dye molecule. Moreover, the least effective initiators in the series are **4** and **14** because of a spherical hindrance caused by a methyl substituent at the ortho position to the methine bond. Generally, the initiators with electron-donating groups for which the free rotation between benzene ring carbon and nitrogen of *N*-alkylamino group is possible indicate a higher rate of heat evolution in comparison to the dyes possessing a stiffened dialkylamino group (see data in Table 2).

**Figure 6:**
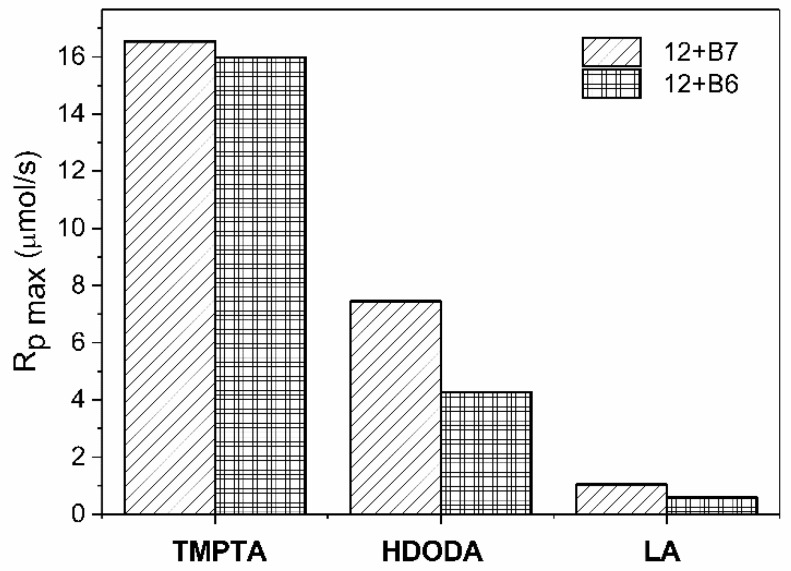
Comparison of the mono-, bi- and tri-functional acrylate monomers polymerization initiated by merocyanine—borate salt pairs. **B7** designation refers to the tetramethylammonium phenyltributylborate and **B6** to the tetramethylammonium phenyltriethylborate applied as co-initiator with the **12** dye. They concentration was 0.05 M and the light intensity was 100 mW/cm^2^.

It is also evident that a slightly better polymerization effect was achieved for tetramethylammonium phenyltributylborate salt used as a co-initiator in the dye photoinitiating system as it is evident from Figure 6. This means that the efficiency of the radical polymerization depends on the alkyl group bound to boron and consequently on the type of the generated radicals and their reactivity. The rate of the radical polymerization was also influenced by the kind of monomer used. The results obtained are in line with the general rules and polymerization is more effective for multifunctional monomers.

Taking into account the general mechanism of initiation of the photopolymerization reaction [4], the photophysical and photochemical processes occurring after absorption of the photon and before the initiating step influence the effectiveness of the reaction. Thus, the reactivity of the tested systems in TMPTA photopolymerization depends on the overall rate of the formation of alkyl radicals, which start a chain reaction, and their reactivity. The formation of boranyl radicals is mainly affected by the ease of the borate salt oxidation, radical bond order, and their stability. The most reactive are alkyl radicals of high stability and lower bond order of a carbon atom with an unpaired electron [19,20]. For the tested systems (**12** + **B6** and **12** + **B7**), in respect to the co-initiator structure, the difference in the initiation efficiency is influenced by substantial differences in the oxidation potentials of the borate salts and the reactivity of the free radicals formed after photoinduced electron transfer process. Since the tetramethylammonium phenyltri-*n*-butylborate (**B7**) is a little more oxidizing than the tetramethylammonium phenyltriethylborate (**B6**), which results from the lower value of its oxidation potential (0.708 V vs. 0.764 V), it generates free radicals more effectively than the phenyltriethylborate salt [19,20]. Moreover, the *n*-butyl radical produced from the NMe_4_B(Ph)*n*-Bu_3_ borate which is more stable than the ethyl radical yielded from NMe_4_B(Ph)Et_3_ is somewhat more reactive in the initiation of the polymerization reaction. It was found that the reactivity order based on the stability of the radicals produced correlates reasonably with the oxidation potentials of the borates studied and the rate of polymerization.

Another crucial parameter that influences the overall reactivity of the tested systems in TMPTA photopolymerization is co-initiator concentration. For identical monomer-dye formulation, a distinct increase in the rate of polymerization from 0.44 to 5.31 μmol/s and from 0.49 to 5.77 μmol/s is observed as the **B6** and **B7** concentrations increase from 0.01 to 0.05 M. This effect relates to a higher amount of alkyl radicals formed after photoinduced electron transfer (PET) (see Figure S4 in Supplementary Materials). 

In addition to the concentration of borate salt, the rate of TMPTA polymerization depends on the optical density (OD) of the dye. It was observed that the polymerization rate was initially proportional to the OD, and consequently the amount of initiator, and then decreased due to “inter filter effect” [49]. This effect occurs when the light is absorbed completely only within a small layer of the sample. This leads to a high local concentration of the radicals and self-quenching process. The most efficient polymerization was observed (for the experimental condition) at the optical density equal to 0.25 (at the excitation wavelength, 488 nm) (see Figure S5 in Supplementary Materials ).

Based on our insights into polymerization initiated by the merocyanine–borate salt systems and literature data for dye-based photoinitiators [4], it is known that irradiation of these two component systems leads to electron transfer from the borate anion to the excited sensitizer. The initiating alkyl radicals are formed from cleavage of the boranyl radical. The electron transfer from the borate anion to the excited dyes is thermodynamically allowed if the free energy (Δ*G_et_*), calculated from the Rehm–Weller equation (Equation (1)) [50], is negative.

(1) $$\Delta G_{et}=E_{ox}(D/D^{•+})-E_{red}(A^{•-}/A)-E_{00}-\frac{Ze^{2}}{\varepsilon a}$$

In our investigation, Δ*G_et_* was calculated using the oxidation potential of the borate salt *E_ox_(D/D^•^⁺)*, the excited state energy *E*_00_, and the reduction potential *E_red_(A^•−^/A)* of the dyes (see Table 2). The Coulombic energy coefficient (*Ze^2^/εa*) was omitted in these calculations because a neutral radical of the borate compound was formed in the electron transfer process. The calculated thermodynamic parameters listed in Table 2 indicate that all the tested dye–borate salt systems possess a favorable thermodynamic driving force (Δ*G_et_* < −0.4 eV) upon exposure to light. These results mean that the photoelectron transfer processes easily occur through the excited state. This, in turn, confirms that the tested dyes in combination with borate salts effectively generate free radicals that can start polymerization of acrylate monomers.

It is apparent that the merocyanine dyes combined with phenyltrialkylborate salts are effective photoredox pairs initiating the polymerization of acrylates. The detailed revision of the data presented in Table 2 indicates that the rate of polymerization might be a function of the rate of the primary process (e.g., the rate of electron transfer within the photoredox pair). The rate of the electron transfer process can be expressed by Equation (2) [4,51,52]:

(2) $$k_{et}=\chi Z\exp(-\Delta G^{\#}/RT)$$

where *Z* is an universal frequency factor (6 × 10^12^ s^−1^) at 25 °C, χ is the transmission coefficient, and Δ*G^#^* is the total energy of activation described by the Marcus equation (Equation (3)) [51]:

(3) $$\Delta G^{\#}=\frac{\lambda }{4}\left(1+\frac{\Delta G_{et}}{\lambda }\right)$$

where *λ* is defined as the total reorganization energy necessary to reach the transition states both of the excited molecule and solvent molecules and Δ*G_et_* is expressed by the Rehm–Weller equation (Equation (1)). 

The logarithmic form of a final equation describing the rate of the photoinitiated polymerization for very viscous or solid monomer including the Marcus relationship is given by Equation (4) [51,53]:

(4) $$\ln R_{p}=A-{\left(\lambda +\Delta G_{et}\right)}^{2}/8\lambda RT$$

where *A* for the initial time of polymerization is the sum: ln*k_p_* – 0.5ln*k_t_* + 1.5ln[M] + 0.5ln*I_a_*. 

**Figure 7:**
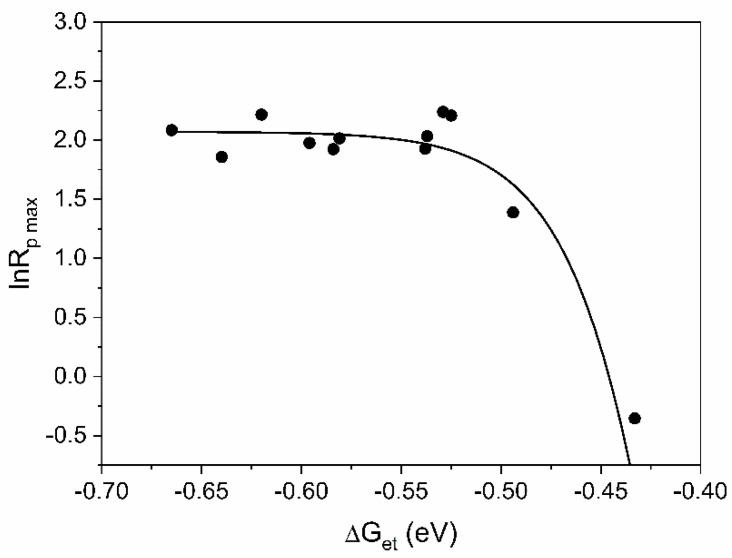
Relationship between the free energy of activation of electron transfer process and the rate of polymerization initiated by the merocyanine dyes and phenyltriethylborate salts.

The experimental verification of Equation (4) is presented in Figure 7. The correlation between the logarithm of the rate of polymerization (ln *R_p_*) and the free energy change (Δ*G_et_*) shows a parabolic relationship. Such a trend is observed if the primary process (e.g., the rate of electron transfer process) controls the rate of photopolymerization.

The plot reveals that the rate of free radical polymerization initiated by the photoredox pairs composed of merocyanine dyes and phenyltrialkylborate salt increases as the driving force of the electron transfer reaction increases. It is noteworthy that such behavior is predicted by the classical theory of the photoinduced electron transfer process [49,50,51,52,53].

## 4. Conclusions

Merocyanine dyes of a A–π–D structure, in which 1,3-dimethylbarbituric acid constitutes an electron-acceptor moiety and 4-alkylaminobenzene serves as an electron-donor group, were tested as a component in the photoinitiating systems for acrylate polymerization. Their linear optical properties were investigated through UV–Vis and fluorescence spectral analysis. Under illumination by laser the tested dyes showed high electron affinity and sensitized phenyltrialkylborate salts to photodecomposition. The sensitization occurred through an electron transfer. These photodecomposition systems may have practical applications as photoinitiators of free radical polymerization of acrylate monomers in visible light. The photoinitiation ability depends on the chemical structure of both the dye and electron donor as well as their concentrations. According to the Marcus relationship, the intermolecular electron transfer process may be the limiting step in the photoinitiated polymerization.

## Figures and Tables

**Table 1:** The photophysical data ^a)^ for compounds **1**–**13**.

Abbr.	Substituent, R	$\;\lambda_{\max}^{Ab}\;(\mathrm{nm})$	$\;\varepsilon_{\max}^{Ab}\;(M^{-1}\;{\mathrm{cm}}^{-1})$	$\;\lambda_{\max}^{Fl}\;(\mathrm{nm})$	$\;\Delta \nu^{SS}\;({\mathrm{cm}}^{-1})$
**1**	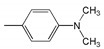	452.5	65,000	521	2906
**2**	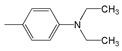	458	86,800	525	2786
**3**	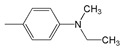	455	77,500	527	3003
**4**	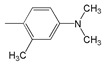	465.5	58,500	539	2929
**5**	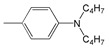	460.5	83,200	532	2919
**6**	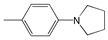	458.5	76,200	529	2907
**7**	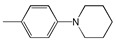	457	61,800	545	3533
**8**	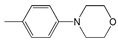	442.5	50,600	521	3405
**9**	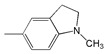	469	61,200	534	2630
**10**	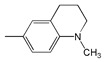	465	78,700	532	2708
**11**	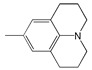	477	93,300	541	2480
**12**	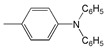	456	40,600	642	6354
**13**	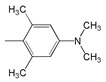	471	20,600	542	2781

^a)^$\lambda_{\max}^{Ab}$ = absorption maxima; $\varepsilon_{\max}^{Ab}$ = maximum extinction coefficient; $\lambda_{\max}^{Fl}=$ fluorescence maxima; $\Delta \nu^{SS}$ = stokes shift of the dyes in ethyl acetate (EtOAc).

**Table 2:** Reduction potential of the merocyanine dyes (*E_red_*), singlet state energy (*E*_00_), free energy of activation for the photoinduced electron transfer (PET) process (Δ*G_et_*), average maximal rates (*R*_p *max av*_), average quantum yields (*Φ_p_*), and average final double bond conversion (*C*^f^_av_) of TMPTA radical polymerization for tested photoredox pairs.

Abbr.	*E_red_* (V)	E_00_ (eV)	Δ*G_et_* (eV)	*R*_p max av_ (μmol/s) ^a)^	*R*_p max av_ (μmol/s) ^b)^	*Φ_p_* ^a)^	*Φ_p_* ^b)^	*C*^f^_av_ (%) ^a)^	*C*^f^_av_ (%) ^b)^
**1**	−1.212	2.641	−0.665	8.04	6.99	98.60	85.73	58	51
**2**	−1.268	2.613	−0.581	7.50	7.08	91.97	86.83	68	55
**3**	−1.276	2.624	−0.584	6.84	6.22	83.91	74.67	57	50
**4**	−1.25	2.508	−0.494	4.01	1.10	49.15	13.47	54	53
**5**	−1.308	2.597	−0.525	9.10	7.04	111.56	86.34	63	53
**6**	−1.298	2.591	−0.529	9.38	7.05	115.05	86.39	65	55
**7**	−1.226	2.528	−0.538	6.88	6.37	84.29	78.14	63	54
**8**	−1.268	2.652	−0.620	9.17	6.88	112.40	84.30	60	53
**9**	−1.168	2.528	−0.596	7.21	4.28	81.62	52.45	54	42
**10**	−1.264	2.565	−0.537	7.64	7.46	93.65	91.51	61	57
**11**	−1.104	2.508	−0.640	6.40	4.78	78.48	58.63	57	54
**12**	−1.224	2.387	−0.399	16.91	15.98	207.37	195.91	73	54
**14**	−1.29	2.487	−0.433	0.70	0.34	8.60	4.22	36	30

^a)^ Optical density of the dye, OD = 0.25; ^b)^ OD = 2.0 with tetramethylammonium phenyltriethylborate (**B6**) as co-initiator.

## Supplementary Materials

The following are available online at https://www.mdpi.com/2073-4360/12/6/1242/s1, Figure S1: Electronic absorption spectra of dye tested in ethyl acetate illustrating the influence of a type of dialkylamino substituent on absorption intensity, Figure S2: Electronic absorption spectra obtained upon irradiation of a system comprising an air-saturated solution of dye tested (1.5 × 10^−5^ M) and borate salt **B6** (1.5 × 10^−3^ M) in ethyl acetate. Spectra collected before and after laser irradiation (line 457 nm, *I*_0_ = 100 mW). Time given in seconds, Figure S3: Exemplar kinetic curves recorded during the measurements of the heat flow emitted during the photoinitiated polymerization of the TMPTA: *γ*-butyrolactone (9:1) mixture initiated by merocyanine dye—borate salt (**B6**) system marked in the figure. The photoinitiator optical density at irradiation wavelength (488 nm) was either 0.25 or 2. **B6** concentration was 0.05 M, light intensity *I*_0_ = 100 mW/cm^2^, Figure S4: Maximal rate of photoinitiated polymerization vs. concentration of the electron donor. The **1** dye concentration was 1 × 10^−3^ M, Figure S5: Maximal rate of polymerization vs. photoinitiator concentration. **B6** concentration was 0.05 M, light intensity 100 mW/cm^2^.
