# Supplementary material for: Highly Effective Sensitizers Based on Merocyanine Dyes for Visible Light Initiated Radical Polymerization
Source: Polymers (Basel). 2020 May 29;12(6):1242. doi: 10.3390/polym12061242 (PMC7361998; doi:10.3390/polym12061242)
Supplement: Supplementary file 1 [file polymers-12-01242-s001.pdf]

Highly Effective Sensitizers Based on Merocyanine Dyes for Visible Light Initiated radical Polymerization

Beata Jędrzejewska[1*], Grażyna Wejnerowska[1]

[1]UTP University of Science and Technology, Faculty of Chemical Technology and Engineering

Seminaryjna 3, 85-326 Bydgoszcz, Poland

**Corresponding author:*

B. Jędrzejewska: e-mail address: beata@utp.edu.pl; Fax: +48 52 374 9009, Tel: +48 52 374 9034

**Table of Contents**	Page(s)
Absorption spectra in ethyl acetate – Figure S1	2
Absorption spectra upon irradiation in ethyl acetate – Figure S2	2–4
Polymerization kinetic curves – Figure S3	4–7
Co-initiator concentration – Figure S4	8
Sensitizer concentration – Figure S5	8

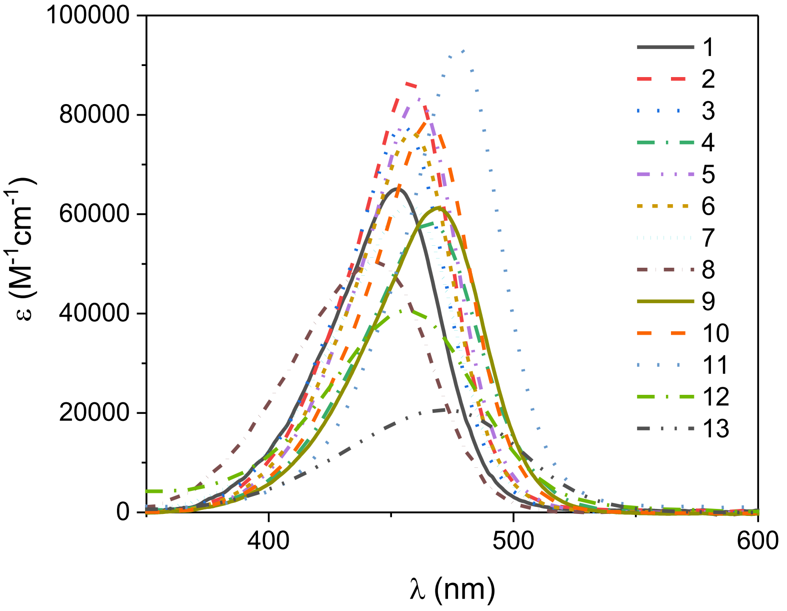

Figure S1. Electronic absorption spectra of dye tested in ethyl acetate illustrating the influence of a type of dialkylamino substituent on absorption intensity.

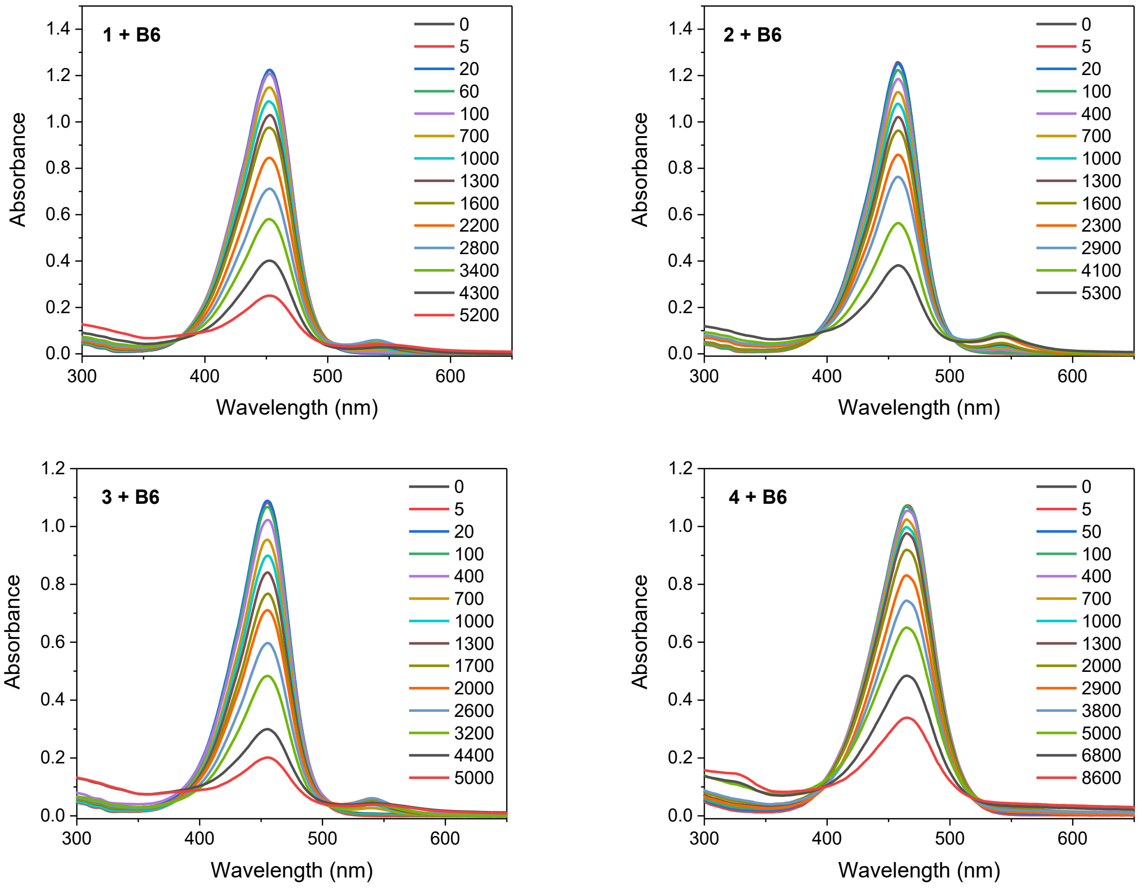

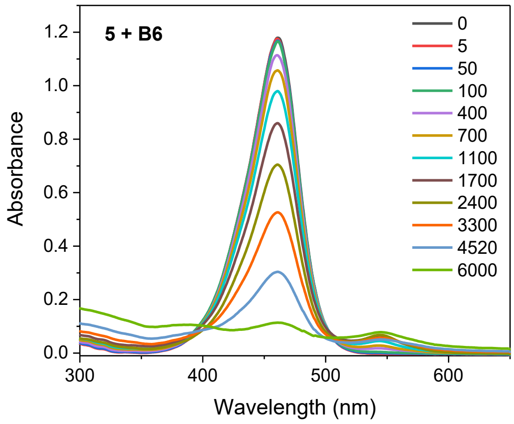
5 + B6
Absorbance
Wavelength (nm)
0
5
50
100
400
700
1100
1700
2400
3300
4520
6000

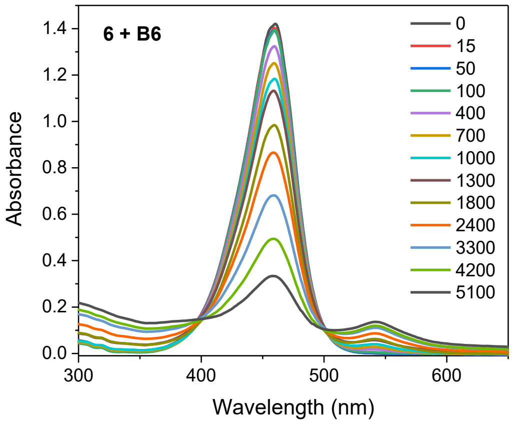
6 + B6
Absorbance
Wavelength (nm)
0
15
50
100
400
700
1000
1300
1800
2400
3300
4200
5100

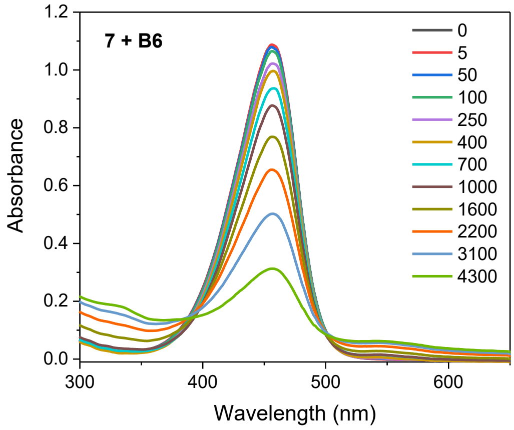
7 + B6
Absorbance
Wavelength (nm)
0
5
50
100
250
400
700
1000
1600
2200
3100
4300

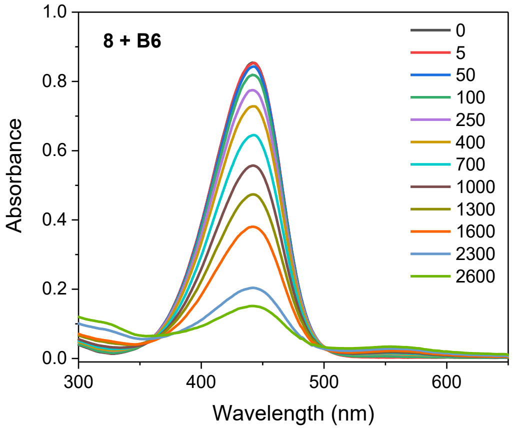
8 + B6
Absorbance
Wavelength (nm)
0
5
50
100
250
400
700
1000
1300
1600
2300
2600

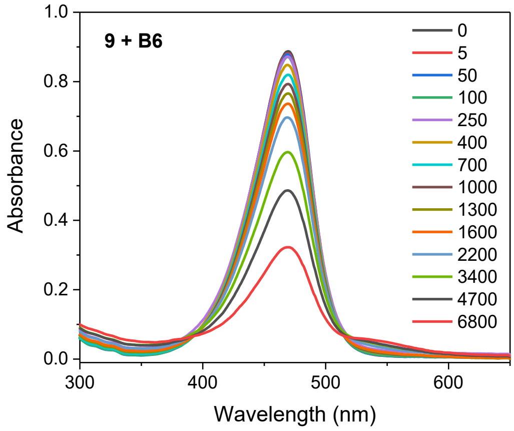
9 + B6
Absorbance
Wavelength (nm)
0
5
50
100
250
400
700
1000
1300
1600
2200
3400
4700
6800

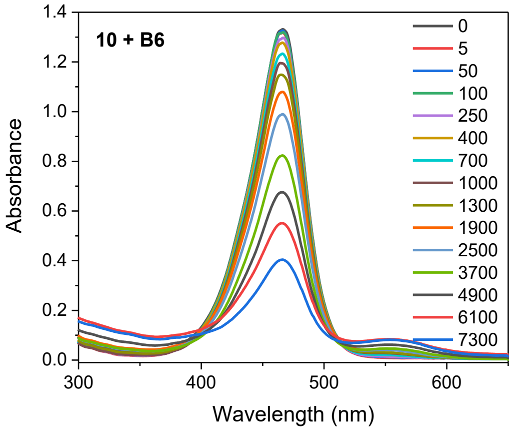
10 + B6
Absorbance
Wavelength (nm)
0
5
50
100
250
400
700
1000
1300
1900
2500
3700
4900
6100
7300

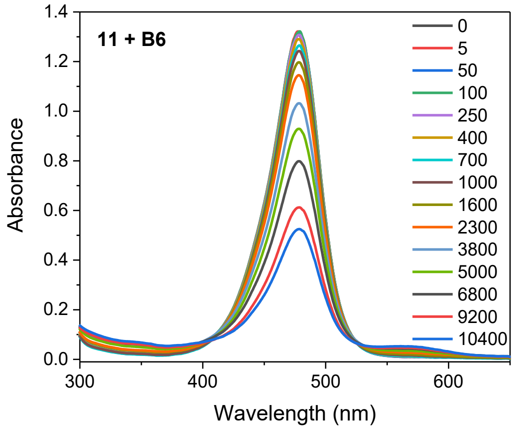
11 + B6
Absorbance
Wavelength (nm)
0
5
50
100
250
400
700
1000
1600
2300
3800
5000
6800
9200
10400

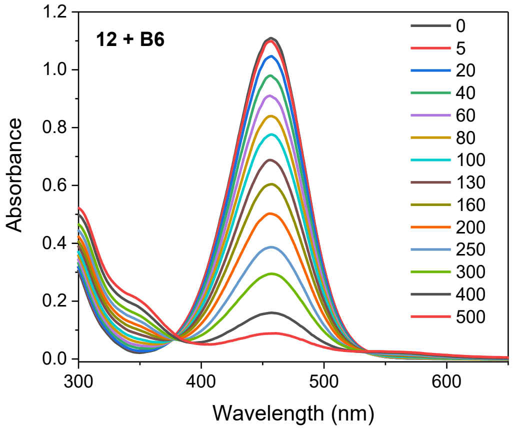
12 + B6
Absorbance
Wavelength (nm)
0
5
20
40
60
80
100
130
160
200
250
300
400
500

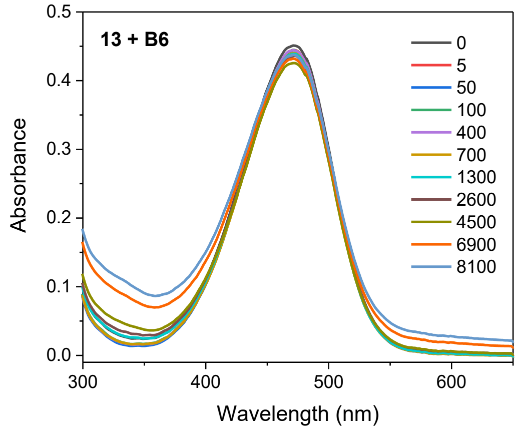

Figure S2. Electronic absorption spectra obtained upon irradiation of a system comprising an air-saturated solution of dye tested (1.5×10^{-5} M) and borate salt **B6** (1.5×10^{-3} M) in ethyl acetate. Spectra collected before and after laser irradiation (line 457 nm, I_0 = 100 mW). Time given in seconds.

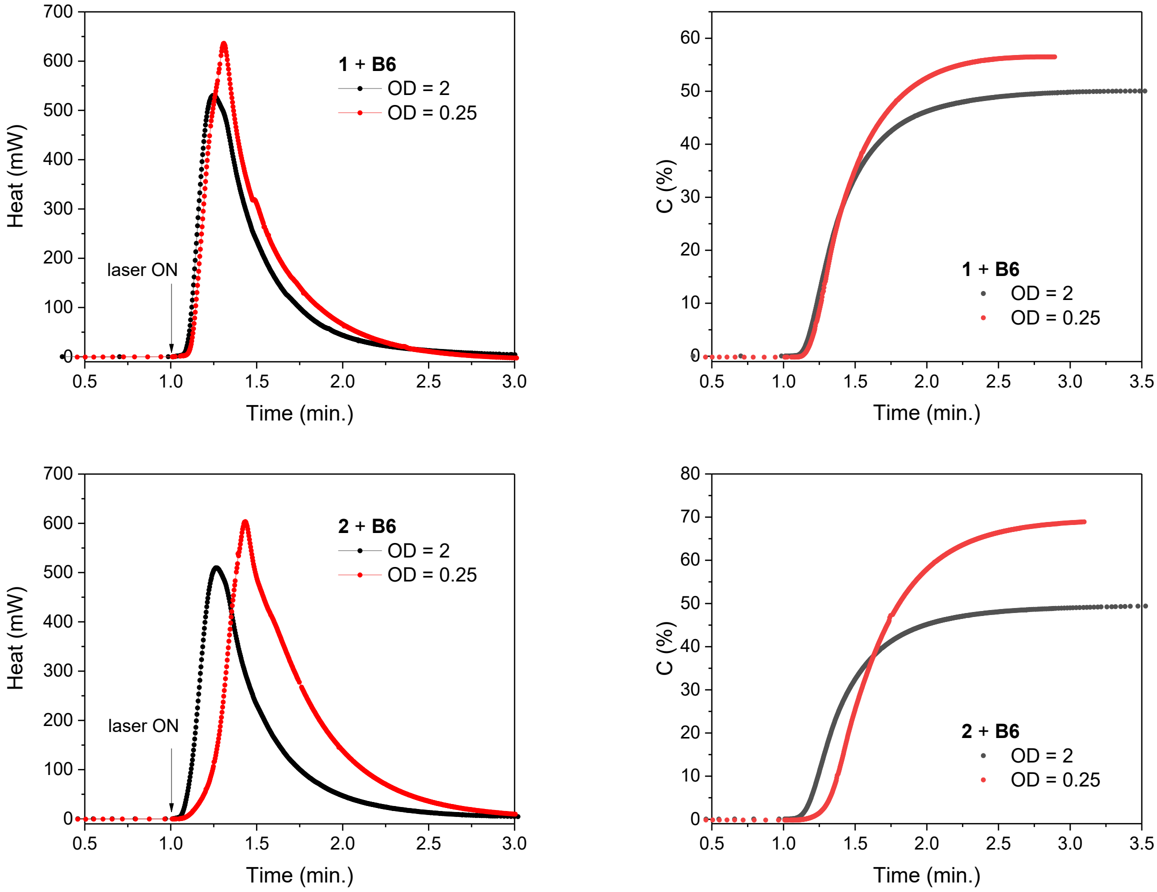

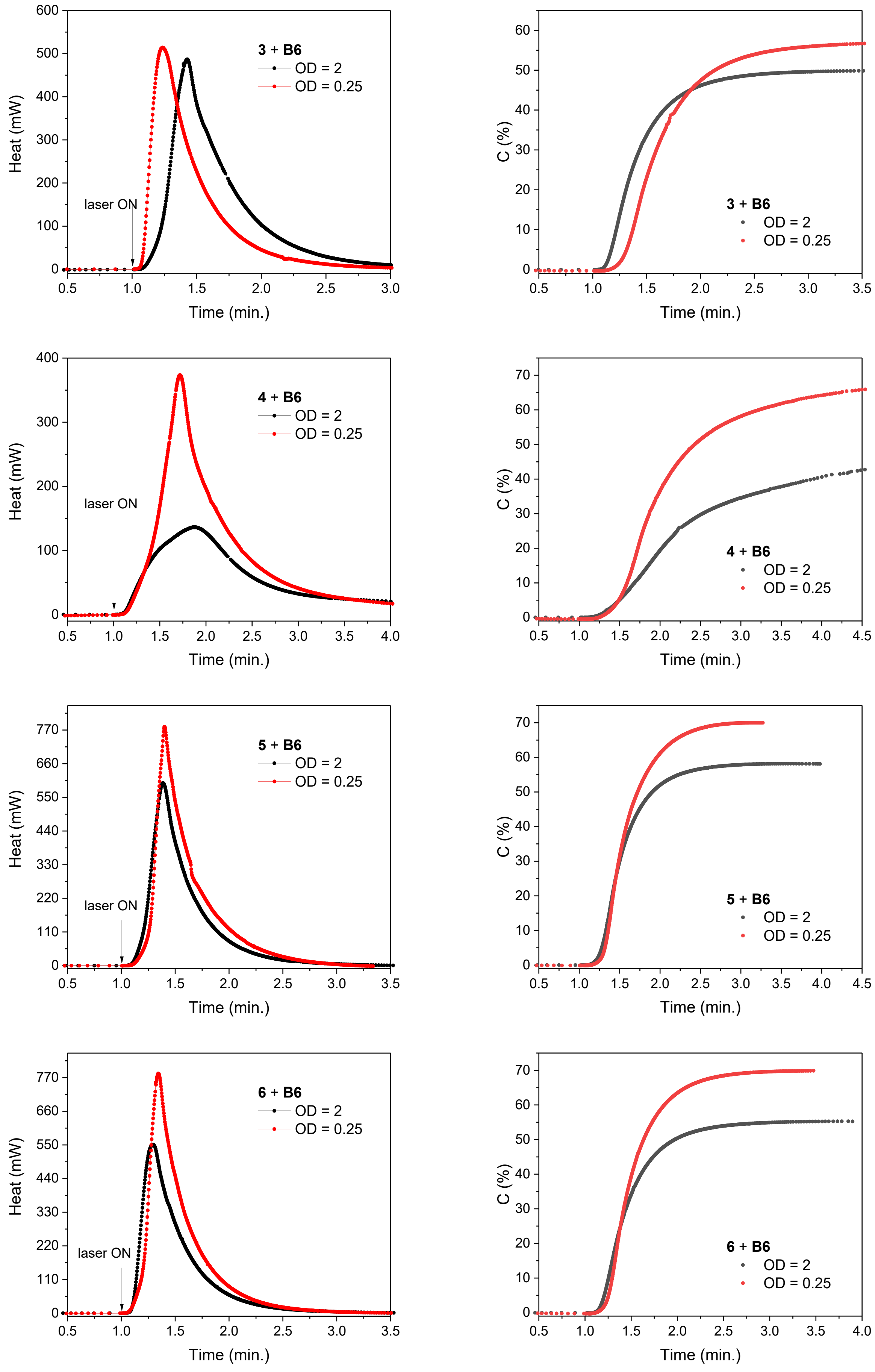
Heat (mW)
Time (min.)
laser ON
3 + B6
OD = 2
OD = 0.25
C (%)
Time (min.)
3 + B6
OD = 2
OD = 0.25
Heat (mW)
Time (min.)
laser ON
4 + B6
OD = 2
OD = 0.25
C (%)
Time (min.)
4 + B6
OD = 2
OD = 0.25
Heat (mW)
Time (min.)
laser ON
5 + B6
OD = 2
OD = 0.25
C (%)
Time (min.)
5 + B6
OD = 2
OD = 0.25
Heat (mW)
Time (min.)
laser ON
6 + B6
OD = 2
OD = 0.25
C (%)
Time (min.)
6 + B6
OD = 2
OD = 0.25

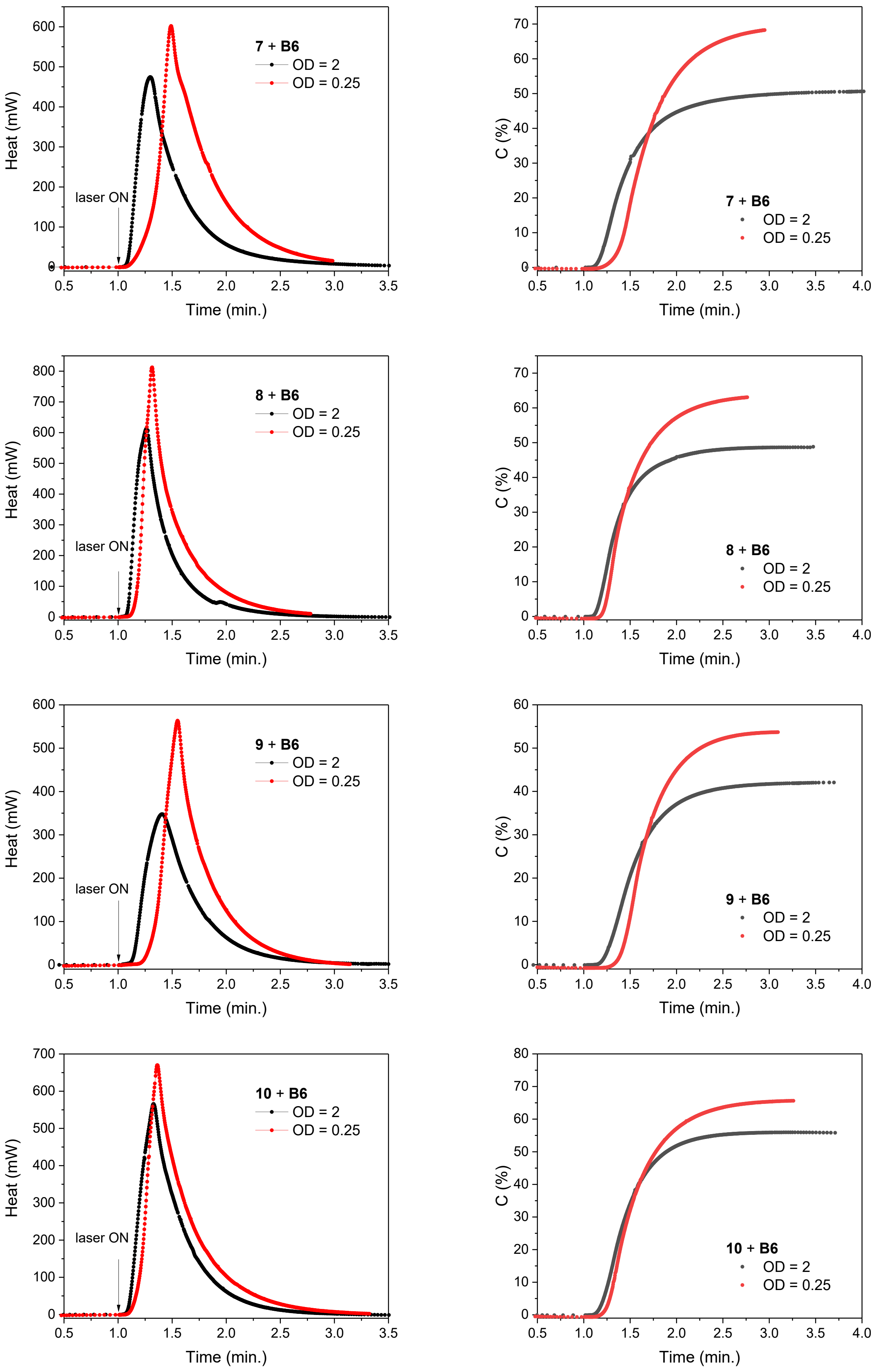

7 + B6
OD = 2
OD = 0.25
laser ON
Heat (mW)
Time (min.)
C (%)
8 + B6
OD = 2
OD = 0.25
laser ON
Heat (mW)
Time (min.)
C (%)
9 + B6
OD = 2
OD = 0.25
laser ON
Heat (mW)
Time (min.)
C (%)
10 + B6
OD = 2
OD = 0.25
laser ON
Heat (mW)
Time (min.)
C (%)

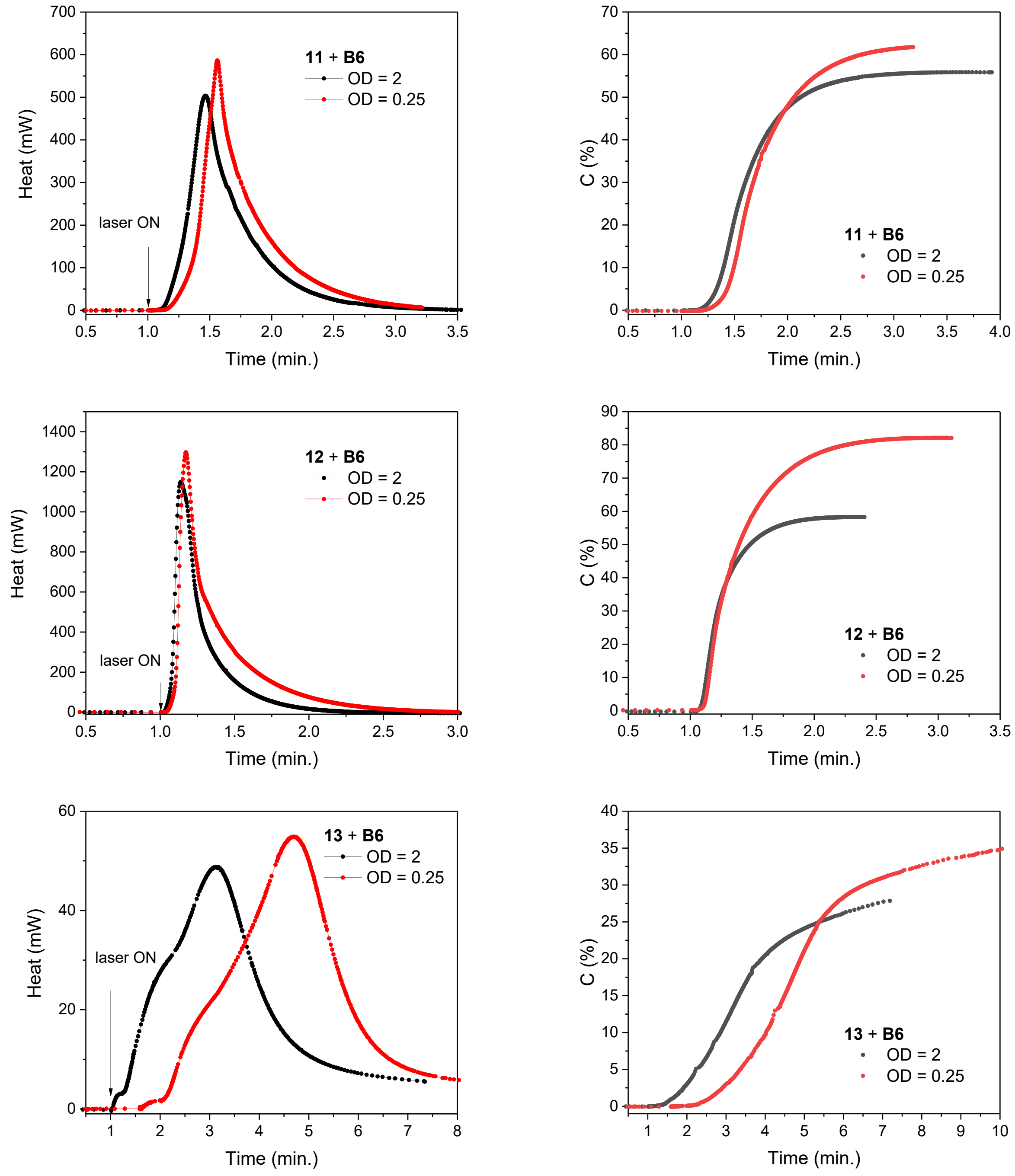

Figure S3. Exemplar kinetic curves recorded during the measurements of the heat flow emitted during the photoinitiated polymerization of the TMPTA:γ-butyrolactone (9:1) mixture initiated by merocyanine dye—borate salt (**B6**) system marked in the figure. The photoinitiator optical density at irradiation wavelength (488 nm) was either 0.25 or 2. **B6** concentration was 0.05 M, light intensity I_0 = 100 mW/cm^2.

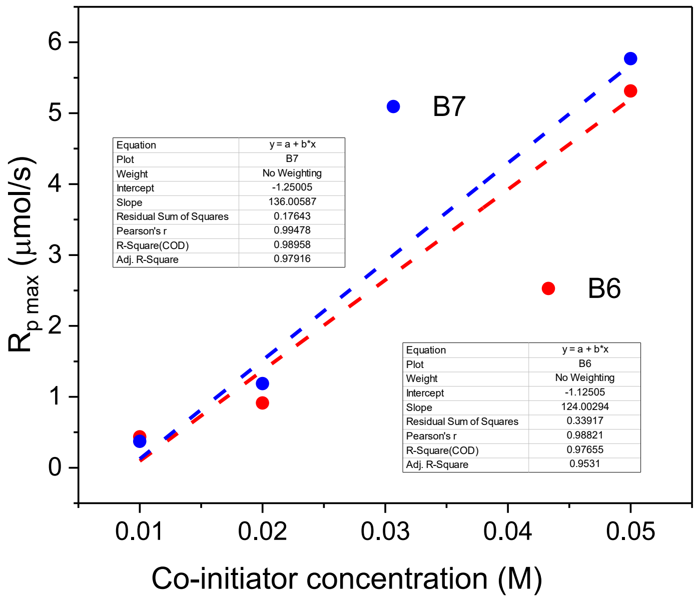

Figure S4. Maximal rate of photoinitiated polymerization vs. concentration of the electron donor. The **1** dye concentration was 1 ×10^{-3} M.

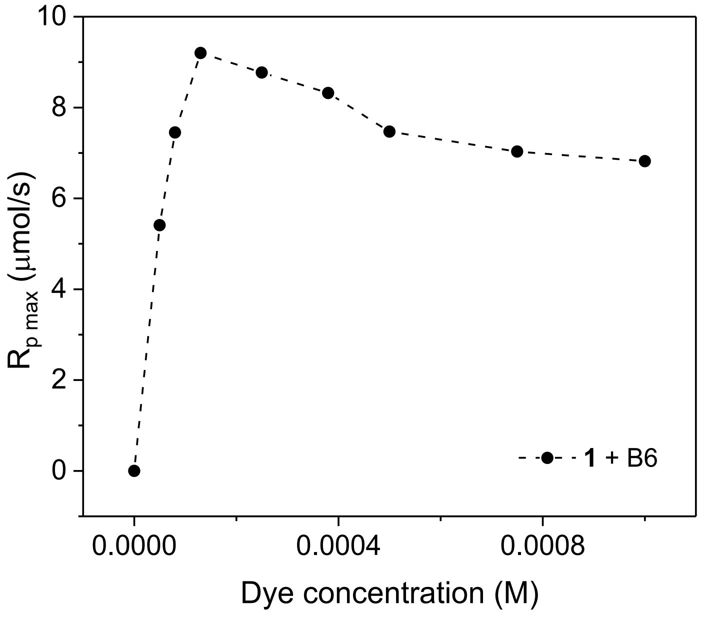

Figure S5. Maximal rate of polymerization vs. photoinitiator concentration. **B6** concentration was 0.05 M, light intensity 100 mW/cm^2.
